# Feasibility and yield of HIV screening among adult trauma patients presenting to an urban emergency department of a tertiary referral hospital in Tanzania

**DOI:** 10.1186/s12981-019-0223-5

**Published:** 2019-04-09

**Authors:** Juma Ramadhani, Hendry R. Sawe, Said S. Kilindimo, Juma A. Mfinanga, Ellen J. Weber

**Affiliations:** 1 Emergency Medicine Department, Muhimbili University of Health and Allied Science, Dar es Salaam, Tanzania; 2 Emergency Medicine Department, Muhimbili National Hospital, Dar es Salaam, Tanzania; 3 Department of Emergency Medicine, University of California San Francisco, San Francisco, CA USA; 4 Emergency Medicine Department, Tanga Regional Referral Hospital, P.O. Box 452, Tanga, Tanzania

**Keywords:** Feasibility, Yield, HIV screening, Trauma, Emergency department, Tanzania, Africa

## Abstract

**Background:**

The World Health Organization and Tanzanian National Guidelines for HIV and AIDS management, recommends provider initiated testing and counseling for HIV at any point of health care contact. In Tanzania, over 45% of people living with HIV are unaware of their HIV positive status. We determine the feasibility and yield of HIV screening among otherwise healthy adult trauma patients presenting to the first full-capacity Emergency Department in Tanzania.

**Methods:**

This was a prospective cohort study of consecutive adult trauma patients presenting to Emergency Medicine Department at Muhimbili National Hospital (EMD-MNH) in Dar es Salaam, from March 2017 to September 2017. Eligible patients provided informed consent, pre and post-test counseling was done. Structured case report forms were completed, documenting demographics, acceptance of testing, results and readiness to receive results. Outcomes were the proportion of patients accepting testing, proportion of positive tests, readiness of the patient to receive the results, and proportion of patients who had an HIV test ordered as part of care.

**Results:**

We screened 2848 trauma patients, and enrolled 326 (11.5%) eligible patients. Median age was 33 (IQR 25–42 years), and 248 (76.0%) of participants were male. Of those enrolled, 250 (76.7%) patients accepted testing for HIV, and among them 247 (98.8%) were ready to receive their test results. Of those tested, 14 (5.6%) were found to be HIV positive and 12 were ready to receive results. Two months post hospital discharge 6 (50%), of those who were informed of positive results had visited Care and Treatment Clinics (CTC) for HIV treatment. Three additional patients had not yet attended and three could not be reached. The treating ED physician tested none of the enrolled patients for HIV as part of their regular treatment.

**Conclusions:**

In our cohort of adult trauma patients presenting to ED, routine HIV screening for unrelated reason, was feasible and acceptable. The yield is sufficient to warrant an on-going program and superior to having physicians choose which patients to test. Future studies should focus on factors affecting the linkage to CTC among HIV positive patients identified at the ED.

## Background

Human Immunodeficiency Virus (HIV) continues to be a major world public health problem, having claimed more than 35 million lives so far. In 2017, approximately 940,000 people died from HIV-related causes globally [1]. Sub-Saharan Africa is the most affected region, with 25.8 million people living with HIV in 2017. It accounts for two-thirds of the global total of new HIV infections [2–4].

Tanzania has about 1.4 million people living with HIV, with adult prevalence of 4.7%. In 2015, 54,000 people were newly infected with HIV mainly between the age group between 15 and 49 years, and 36,000 people died from an AIDS-related illness in the same year [5, 6]. Of all people living with HIV at the moment globally, it is estimated that only 60% know their status. The remaining 40% or over 14 million people need to access HIV testing services [7]. The World Health Organization (WHO), and Tanzanian National Guideline for Management of HIV and AIDS recommend routine HIV testing in all health facilities [8, 9]. Through provider initiated testing and counselling, people living with HIV might be identified early and can then be enrolled into the management system [10].

Routine HIV screening testing at the emergency department (ED) provides an opportunity for early recognition of HIV positive patients [11, 12]. Identifying them early will provide the possibility of initiating treatment early so as to stop the progression to AIDS. Further more, the identification of HIV negative individuals will provide an opportunity of linkage to HIV prevention services. Studies in other countries have shown that the routine HIV testing at the ED is feasible with a very good acceptance rate and with good yield [12, 13]. Those diagnosed HIV positive have been enrolled in ongoing follow-up for their continuous care [14]. However, as a emergency medicine is very new field in Tanzania, there has been no study done in Tanzania looking the feasibility of routine HIV testing and its yield in emergency departments. The first full capacity Emergency Department was opened in 2010 in at the main referral hospital in Dar es salaam. In this ED, HIV testing occurs on a provider-initiated basis, but it is generally performed only on medically ill patients for the diagnostic purpose, and not as part of the routine ED Screening. The study aimed to determine the feasibility and yield of HIV screening among otherwise healthy adult trauma patients as these trauma patients present non-medically ill individuals who would otherwise not have attended hospitals and would therefore not have opportunity to have an ED provider initiated testing. With the goal towards assessing the possibility of creating an ED protocol that would allow every patient attending the ED in Tanzania to be counseled for taking the HIV test.

## Methods

### Study design

This was a prospective cohort study of adult trauma patients presenting to the Emergency Medicine Department of Muhimbili National Hospital from March 2017 to September 2017.

### Study area

Muhimbili National Hospital (MNH) is a tertiary referral hospital located in Dar es salaam, Tanzania. The hospital has a bed capacity of 1500 with around 1000 to 1200 admissions per week, operating 24 h in 7 days of the week. The MNH-EMD is the first public ED in the country and was opened in 2010. It is staffed with emergency physicians, postgraduate students in emergency medicine training program, medical officers, critical care nurses and nursing officers. The MNH-EMD is the first entry point to the hospital for most of the patients attending MNH, and sees around 200 patients daily. Acutely ill patients are received at ED, resuscitated and stabilized before being disposed to the appropriate ward. Currently HIV testing at the ED is based on physician discretion and is usually done on only sick medical patients.

### Participants

All adult (≥ 18 years old) trauma patients attending the EMD-MNH were eligible for the study. Patients were excluded if they had altered mental status, could not speak English or Kiswahili, those who are known to have HIV infection as evidenced by an HIV treatment card, pregnant women already attending Antenatal clinics, and hemodynamic instability—as defined by the treating physician.

### Study procedures

The research assistant frequently and actively went through the entire department (resuscitation rooms and low acuity areas) during 12 h of day clinical shifts of week days to identify trauma patients with the help of nurses in the respective area. The patient registry was also reviewed to identify trauma patients present the department.

All adult trauma patients meeting the inclusion criteria were offered a chance to participate in the study after they had received initial treatment and stabilization. The research assistant explained the study, and patients reviewed and signed a consent form to be involved in the study (Consent for the study did not mean consent for testing.) Participants then received pre- testing HIV counseling provided by an EMD nurse who had training in HIV counseling and testing. Participants were informed that no extra charges would be incurred for the test, and that their treatment plan will not be affected by their decision to be tested. Patient privacy and confidentiality were carefully observed before and after testing regardless of the test results.

Structured case report forms were completed, documenting demographics, acceptance of testing, results and readiness to receive results.

### Testing

Testing was done as per the 2016 National Guidelines for HIV and AIDS Management in Tanzania. Finger prick blood sample was collected then tested in the EMD for HIV using rapid immunochromatographic assay (*SD*-*BIOLINE HIV*-*1/2 Alere, Abbott Laboratories, Illinois USA*) as the first testing tool. Those with a negative SD BIOLINE test were considered free of HIV. If the *SD*-*BIOLINE HIV*-*1/2* test positive, a second gold standard immunochromatographic (*Uni*-*Gold™ HIV, Trinity Biotech Plc, Ireland*) test was used as the confirmatory test. Those with *Uni*-*Gold HIV* test positive were considered to have HIV infection. A negative *Uni*-*Gold HIV* test was considered inconclusive; in this case the blood sample was taken to the central pathology laboratory for HIV antibody and antigen testing and results were received the same day.

### Post testing counseling

Post testing counseling was done before test results were provided. Patients were asked about their readiness to receive the test result. For those who were ready to receive test results, the results were provided. Those who were not ready to receive the test results were counseled to come back to the department at any moment that they felt ready to receive the test results, and were also told they can always go to the nearby health facility of their choosing to be tested for HIV again whenever they are ready. Those who were willing to receive their results and were positive for HIV were provided a special referral form and instructed to attend Counseling and Treatment Centers (CTC) and other HIV treatment centers for further assessment and care after their discharge from the hospital.

### Provider-selected testing

For each enrolled patient, the Wellsoft electronic health record (Wellsoft Corporation, Somerset, USA) was reviewed to determine if the treating physician had ordered an HIV test. The proportion of individuals tested for HIV, and positive cases detected by the EMD physician, were compared with that detected by routine testing during the study.

### Feasibility

Feasibility was assessed by noting any problems with availability of the test, difficulty of administering the test or assessing results, or any adverse events.

### Follow up

HIV positive patients were contacted by telephone 2 months after their discharge to ask if they had made a visit to CTC as they were instructed.

### Data analysis

Data from *REDCap* (*Version 7.2.2, Vanderbilt, Nashville, TN, USA*) was exported to Statistical Package for Social Science (SPSS version 22.0 IBM Ltd, Carolina, USA) for analysis. Descriptive statistics; median, proportion, interquartile range [IQR] and counts are reported. Proportion of trauma patients accepting HIV testing was calculated as enrolled adult trauma patients who accepted testing divided by the total number of adult trauma patients enrolled in the study. The yield was the proportion positive tests among those who accepted testing and was calculated by dividing the adult trauma patients who tested positive for HIV by the total number of adult trauma patients who accepted testing.

## Results

**Fig. 1:**
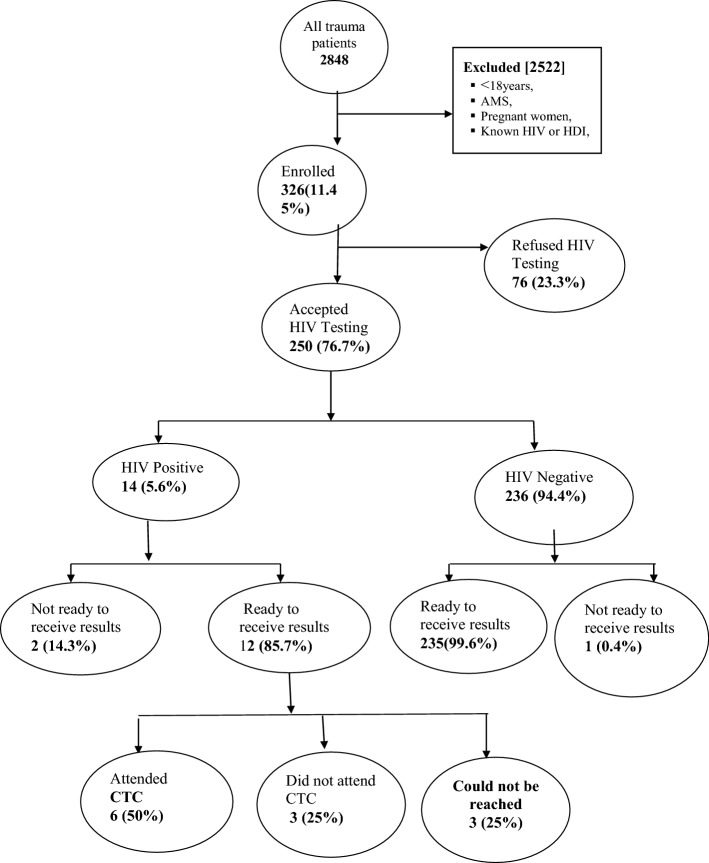
Study Enrollment

During the study period 2848 trauma patients presented to the EMD, among them 326 (11.45%) met the inclusion criteria, gave written consent and hence enrolled in the study (Fig. 1).

### Demographic characteristics

A total of 326 adult trauma patients were enrolled, Median age was 33 (IQR 25–42 years) and 248 (76%) were male. Total of 136 (48%) were referred from other hospitals to MNH. Approximately half of patients (160) were married, 173 (53%) were unemployed and 224 (69%) had no formal education beyond primary school Table 1.

**Table 1:** Demographic characteristics of trauma patients approached for an opt out HIV screening attending at EMD of MNH

Variable	Frequency, N (%)
Sex	
Male	248 (76%)
Female	78 (24%)
Age groups	
Median age (IQR)	33 (25–42)
18–31	145 (44%)
32–41	100 (31%)
42–53	51 (16%)
54–66	19 (6%)
> 66	11 (3%)
Referral status	
Referred	136 (48%)
Not referred	190 (58%)
Marital status	
Married	160 (49%)
Single	154 (47%
Divorced	5 (2%)
Widowed	7 (2%)
Occupation	
Public or private servant	102 (31%)
Unemployed	173 (53%)
Petty trader	32 (10%)
Student	15 (5%)
Others	4 (1%)
Education level	
Primary education	224 (69%)
Secondary education	73 (22%)
College/University education	21 (6%)
No formal education	8 (3%)

### Acceptance and readiness to receive test results

Of 326 patients enrolled and counseled for HIV testing, 250 patients (77%) accepted testing; 69% of all males accepted testing while all females (100%) accepted testing. Married and single patients were similar in their willingness to be tested. Acceptance was high (79%) among those with primary education.

Among the 250 patients tested, 247 (98.8%) were ready to receive their test results. One male and two females were not ready to receive their HIV test results; all 3 were single, and had not gone beyond primary school. All three had been referred from other hospitals to MNH (Tables 2 and 3).

**Table 2:** Distribution by gender, age, marital status and referral status of trauma patients who accepted HIV screening and were ready to receive the test results

Variable	Number	Accepted Testing for HIV	Ready to receive test results	Test results positive
Overall	N	n/N (%)	n/N (%)	n/N (%)
Total	346	250 (77.0%)	247 (98.8%)	14 (5.6%)
Sex				
Male	248	172 (69.0%)	171 (99.0%)	9 (5.2%)
Female	78	78 (100%)	76 (97.0%)	5 (6.4%)
Age group				
18–31	145	109 (76%)	107 (98%)	5 (4.6%)
32–41	100	78 (78%)	77 (99%)	5 (6.4%)
42–53	51	41 (80%	41 (100%)	1 (2.4%)
54–66	19	15 (79%)	15 (100%)	2 (13.3%)
> 66	11	7 (64%)	7 (100%)	1 (14.3%)
Marital status				
Married	160	127 (79%)	127 (100%)	5 (3.9%)
Single	154	115 (75%)	112 (97%)	8 (7.0%)
Divorced	5	3 (60%)	3 (100%)	0
Widowed	7	5 (71%)	5 (100%)	1 (20.0%)
Referral status				
Referred	190	152 (80%)	149 (98%)	12 (7.9%)
Not referred	136	98 (72%)	98 (100%)	2 (2.0%)

**Table 3:** The distribution by occupation and education level of trauma patients who accepted the HIV screening and were ready to receive the test results

	Number	Accepted testing for HIV	Ready to receive test results	Test results positive
*Occupation*				
Public or private servant	102	77 (75%)	75 (97%)	6 (7.8%)
Unemployed	173	137 (79%)	136 (99%)	6 (4.4%)
Petty trader	32	24 (75%)	24 (100%)	2 (8.3%)
Student	15	8 (53%)	8 (100%)	0
Others	4	4 (100%)	4 (100%)	0
*Education level*				
Primary education	224	176 (79%)	173 (98%)	14 (8.1%)
Secondary education	73	57 (78%)	57 (100%)	0
College/university education	21	12 (57%)	12 (100%)	0
No formal education	8	5 (63%)	5 (100%)	0

### Yield

Out of 250 patients who agreed to be tested, 14 (5.6%) tested positive for HIV 9 (5.23%) were male and 5 (6.41%) were female (Fig. 1, Tables 2 and 3).

### Self-reported CTC attendance

Among the 12 HIV positive patients that received results, 6 patients (50%) reported having made visits to CTC and were on ARVs; 3 patients (25%) reported to have not gone to CTC. The remaining 3 (25%) were could not reached; either their phone number were not reachable, or the person answering stated it was the wrong number (Fig. 1).

### HIV testing as part of regular ED care

Of 250 patients tested for HIV during the study, none were tested for HIV as part of their regular care in the emergency department. Therefore, all 14 HIV positive patients would not have been identified by the physician who was providing care for these trauma patients.

### Feasibility

During the entire period of study, all patients who agreed to be tested received the test as per National guidelines. The testing kits were available whenever needed, results were provided on time and there were no adverse events throughout study period.

## Discussion

In this study we found that routine HIV testing in an emergency department in Tanzania is feasible and acceptable, majority of patients in the study were willingly tested and ready to receive their results. We found an overall HIV positivity rate of 5.6% among patients who participated in the study.

The majority of enrolled patients were young. This was due to the fact that the study was confined to trauma patients. Younger age people are at greater risk of having trauma because of their daily activities [15, 16]. Previous studies have also shown that younger age groups are also more likely to have HIV [13, 17]. However, we chose trauma patients for this study primarily because we wanted to test the feasibility and acceptability of a routine HIV testing in an otherwise healthy population and compare the results with what providers in our department normally do regarding HIV testing in such patients.

We found that routine HIV screening at the EMD for diagnosing people living with HIV, but unaware of their status is feasible, as evidenced by more than 75% of those counseled agreeing to be tested. Mass education and training has helped to raise the awareness of HIV and AIDS in Tanzania and that likely contributed to a good acceptance rate. While a previous emergency department study in the US have shown a high overall acceptance rate as in our study [23], this is not always the case. A study done in Singapore showed a very low acceptance rate which was 21% [18]. All female trauma patients approached for routine HIV testing accepted; similar studies done in different health care settings in different parts of the world have shown that the acceptance rate tends to be higher in women [17]. In our study, the unanimous acceptance rate by women may be due to the fact that women have more visits to health facilities, including antenatal and postnatal clinics where HIV education is commonly provided.

Almost all trauma patients who accepted testing were ready to receive their test results. This is similar to studies in other sub-Saharan countries: In Uganda, only 4% of patients did not appear for their HIV test result [19]. Another study from Zambia showed that all patients who accepted HIV testing were ready to receive their test results [17]. A study done in Uganda showed that of all those who agreed to be tested, only one person did not return for test results [14]. Even among those that tested positive, we found that more than 85% of those tested positive for HIV were ready to receive their test results. Similarly, studies done in Uganda did not show any difference in readiness to receive test results among those who tested positive for HIV and those who tested negative [19, 20]. This readiness found in all these studies, including ours, was probably due to proper pre- and post-test counseling.

Of those patients that accepted HIV screening, almost 6% tested positive which is similar to HIV prevalence in the country; the Tanzanian National Guidelines for HIV and AIDS management reports the HIV prevalence to be 4.8% [21]. The yield was much higher than in similar studies done in emergency departments in USA [13, 22]. Another study done in Singapore showed the yield to be much lower than in the this study (0.18%) [18]. And a study in the USA found that routine testing identified 1.9% new cases of HIV [23].

In our study, the proportion of positive HIV tests was slightly higher nationally the prevalence of HIV infection in Tanzania in women is 6.3% and 3.9% for men [8]. The higher prevalence of HIV in females is thought to be due to biological differences, which increase the chance of females acquiring the virus. This study did not show a very large difference between males and females, but not all males agreed to be tested.

One of the important goals for ED HIV screening is to link newly HIV diagnosed patients to specialized HIV centers (CTC) for continuous care. In this study, 50% of those who were HIV positive and received their results went to CTC, and at follow up reported they were already on treatment. Three others who were reached (25% of positive results) did not go to CTC. This may be due to the fact that the study enrolled trauma patients and they might not have recovered fully, preventing them from attending CTC. However, similar findings were reported in the study done in United States; only 56% of newly HIV diagnosed patients attended the specialized centers for HIV [13]. Other studies in similar settings in Uganda, Zambia and in Singapore have shown that most of the patients who have tested positive in routine HIV or PITC adhered to instructions to enroll in a clinic [14, 17, 18].

In our department, most HIV screening is done to assist with diagnosis of a medical disease, and so non-suspicious patients rarely get tested. In this study we found that none of the patients that were included in the study would have been tested by the treating physician and so none of the new cases would have been identified. A study of routine HIV testing done in Emergency Department in USA showed that out 9 patients diagnosed with HIV, only 2 of them would have been diagnosed by the physician [13]. In another study conducted in USA most patients with newly diagnosed cases of HIV identified in the ED through routine testing were not admitted to the hospital, and thus would not be identified in a regular hospital care [23].

### Limitations

This was a single center study, which reduces its generalizability. The study excluded children, pregnant women, unconscious patients and those with hemodynamic instability. Three of the positive patients were could not be reached, and three did not receive result limiting the ability to determine compliance with follow up care.

## Conclusion

Routine HIV screening among adult trauma patients being seen in an ED in Tanzania is feasible, acceptable and helps to identify new cases of HIV positive patients who can then enroll for continuous HIV and AIDS care. Future studies should focus on factors that can increase acceptance of testing, and improve the linkage to CTC among HIV positive patients identified at the ED.
